# Effect of vitamin D3 seasonal supplementation with 1500 IU/day in north Italian children (DINOS study)

**DOI:** 10.1186/s13052-018-0590-x

**Published:** 2019-01-28

**Authors:** Stefano Mazzoleni, Giovanna Magni, Daniela Toderini

**Affiliations:** 1 Primary Care Paediatrician Azienda ULSS 6 Euganea Regione Veneto, Polistudio Pediatrico, via D’Annunzio 3/A, Piove di Sacco, Padova, Italy; 2 Senior Biostatistician, NRC Azienda Ospedaliera Padova, Padova, Italy; 3 Unità di Ricerca Clinica, Istituto Oncologico Veneto, Padova, Italy; 4 Endocrinologist and General Practitioner Azienda ULSS 6 Euganea Regione Veneto, Studio via Benizzi 6, Padova, Italy

**Keywords:** Vitamin D, Vitamin D3, Cholecalciferol supplementation, 25-hydroxycholecalciferol, Hypovitaminosis D, Vitamin D deficiency, Vitamin D insufficiency, Seasonal variation, Paediatric primary care, Family paediatrician

## Abstract

**Background:**

The appropriate dose of vitamin D supplementation in children is still debated. We calculated that the recommended dose of 600–1000 IU vitamin D3/day is not sufficient to reach a serum 25-hydroxyvitamin D (25-OH-D) level of at least 30 ng/ml (75 nmol/l) in north Italian children > 12 months. The aim of this study was to analyse the effect of seasonal supplementation with 1500 IU (=37.5 μg) vitamin D3/day.

**Methods:**

DINOS (D-vitamIN Oral Supplementation) study was a pilot, monocentric, non-random case-control register study. It was conducted in a paediatric primary care setting near Padova (North Italy, 45°N latitude). The data of 203 children (girls:boys 1:1,33) aged 2–15 years, collected between November 2010 and January 2015, were analysed. Active group A (*n* = 82) were given 1500 IU vitamin D3/day from November to April; control Group B (*n* = 121) received no supplementation. The serum 25-OH-D test was part of a laboratory tests panel and performed using a chemiluminescence immunoassay method.

**Results:**

Serum 25-OH-D mean level + standard deviation throughout the period was 32 + 13 ng/ml (80 + 32 nmol/l) in group A vs 22 + 10 ng/ml (55 + 25 nmol/l) in group B. In group A 12% had vitamin D deficiency 25-OH-D < 20 ng/ml (50 nmol/l) and 1.2% severe vitamin D deficiency 25-OH-D < 10 ng/ml (25 nmol/l). In group B 46% had vitamin D deficiency and 9% severe deficiency (*P* <  0.001). In group A mean levels were normal or near-normal all the year except in May. Group B reached mean 25-OH-D levels close to 30 ng/ml (75 nmol/l) only in late summer. The active group mean 25-OH-D level was normal in preschoolers and schoolers but not in adolescents. Non-white children had a three-times vitamin D deficiency probability despite supplementation.

**Conclusions:**

Vitamin D supplementation with at least 1500 IU vitamin D3/day from November to April was found appropriate for children in North Italy. A prolongation until May could be useful. Higher doses and/or prolonged periods could be more appropriate especially in adolescents and in non-white children.

**Study registration:**

DINOS gained the approval of Padova Ethics Committee (n. 3960/U16/2016).

**Electronic supplementary material:**

The online version of this article (10.1186/s13052-018-0590-x) contains supplementary material, which is available to authorized users.

## Background

Hypovitaminosis D is highly prevalent worldwide in children and adolescents [1–3], as well as in adults [4]. There are several studies on vitamin D status of children living in North Italy [5–13] but only one was an intervention study, using vitamin D3 (cholecalciferol) [11], and one in paediatric primary care setting [9]. In this research severe vitamin D deficiency (25-OH-D < 10 ng/ml) was rare as in other studies [14–17], but subclinical vitamin D deficiency or insufficiency frequently occurred, as previously described in otherwise healthy people [12, 14, 18, 19].

Various sources recommend 600 IU (1 IU = 25 ng) vitamin D per day for children between 1 and 17 years [20–22]. A recent publication of the Italian Health Minister states that in schoolchildren and adolescents a supplementation is highly recommended if adequate dietary intake and sufficient sun exposure are not guaranteed [23]; this paper suggests an estimate dose of 400–1000 IU per day. The Italian 2015 Consensus recommends 600–1000 IU/day as prophylaxis in children aged 1–17 years at risk of hypovitaminosis D [24].

A recent study on Italian children with moderate vitamin D deficiency (25-OH-D 11–20 ng/ml) demonstrated that a dose of 400 IU vitamin D3/day is insufficient to restore adequate 25-OH-D levels [11]. Assuming that most children living in our research area had less or much less than the minimum desirable level of 25-OH-D [9] and considering that for every supplementary 100 IU/day we can expect an increase of 0.5–1.5 ng/ml in the serum level of 25-OH-D [11, 25–32], we considered that the recommended doses of 600–1000 IU/day would offer no benefit to children with 25-OH-D < 10 ng/ml and even < 20 ng/ml. On this theorical basis, given the high probability of having insufficient or deficient children, to reach the minimum desirable level in most children we decided to prescribe our patients > 12 months 1500 IU (= 37.5 microg) vitamin D3/day from November to April. This dose corresponds to Holick’s suggestion [33] and is about half of the tolerable (safe) upper limit for vitamin D stated by the IOM Committee: 2500 IU/day for children aged 1–3 years, 3000 IU/day for 4–8 years, 4000 for 9 years or older [20]. The European Food Safety Authority (EFSA) has stated a tolerable upper intake level of 2000 IU/day for children from 1 to 10 years of age and of 4000 IU/day for children from 11 to 17 years [34]. The ESPGHAN Committee on Nutrition agreed with this statement [35].

## Methods

### Patients and methods

DINOS (D-vitamIN Oral Supplementation) study is a pilot, monocentric, non-random case-control register study on vitamin D3 1500 IU/day versus no supplementation. Data of over 1000 children till 15 years of age living in a rural area near Padova (Italy, 45°N latitude) were made available for analysis in the electronic medical record system of a primary care paediatrician (PCP, or family paediatrician). In Italy a PCP provides comprehensive and continuing free health care to children, ordinarily from 0 to 14 years of age, as recommended by the European Academy of Paediatrics [36]. All children having at least one serum 25-OH-D measurement between November 2010 and January 2015 were identified; the 25-OH-D test was part of a panel of laboratory tests prescribed for various clinical reasons (such as suspected anaemia, poor growth, fatigue, etc).

### Inclusion criteria

1. age 2–15 years, and
2. 1500 IU vitamin D3/day from November to April (active group A) or no vitamin D supplementation over the last 12 months (control group B), and
3. written consent to the study

### Exclusion criteria

1. any disorder known to affect bone metabolism
2. use of medications inactivating vitamin D
3. other doses of vitamin D3 than 1500 IU/day
4. age < 24 months
5. serious disease potentially interfering with the smooth running of the study.

All eligible children (fulfilling all the mentioned inclusion criteria and having no exclusion criteria) were consecutively enrolled in the study. They were 203 children aged 2–15 years (girls:boys 1:1,33), representing about 20% of those registered in the medical record system. Group B children (***n*** = 121), enlisted since November 2010, had not received any vitamin D supplementation because at that time there was no official Italian NHS vitamin D policy for children older than 1 year. Group A children (***n*** = 82), enlisted since October 2012, had received 1500 IU vitamin D3/day from November to April.

Considering that data from different years were collected, we monitored solar radiation in our area (www.arpaveneto.it).

The used dose of vitamin D3 supplementation was within safety range suggested by scientific societies or authorities [20, 34, 35]. An oral olive oil solution (vitamin D3 10,000 IU/ml, 1 drop = 250 IU) was used.

Parathyroid hormone (PTH), alkaline phosphatase, calcium and phosphorus tests were done only in a few children because the purpose of the study was to analyse the effect of supplementation with 1500 IU vitamin D3/day on vitamin D status and not biochemical consequences of hypovitaminosis D.

All blood samples were obtained between 07:30 am and 09:00 am after an overnight fast. The serum level of 25-OH-D was measured using a chemiluminescence immunoassay method. The laboratory normal minimum was 30 ng/ml (ng/ml × 2.496 = nmol/l), as suggested by several scientific societies [37–41]. Values < 30 ng/ml were considered as hypovitaminosis D. Insufficiency was considered between 20 and 30 ng/ml (this extreme excluded) [39], deficiency as < 20 ng/ml, severe deficiency as < 10 ng/ml [20, 35, 39]. Levels above 100 ng/ml were considered as hypervitaminosis [42]. Vitamin D intoxication is observed with levels > 150 ng/ml [35, 42, 43].

Collected data include age, sex, ethnicity, skin colour, body mass index (BMI) and income. None of the children were vegetarian. In Italy shelf food is generally not fortified with vitamin D. Groups A and B were divided into ages 2–5 (preschoolers), 6–10 (schoolers) and 11–15 years (adolescents). BMI was calculated using the formula weight/square height (kg/m^2^). Children were divided into four categories considering their age and sex percentile according to the method of Cole and Green [44], the Italian growth charts [45] and the WHO definition of underweight [46]: underweight (< 5th percentile), healthy weight (5th < 85th percentile), overweight (85th < 95^th^ percentile), obese (> 95th percentile).

Low-income families were defined as those exempt from NHS prescription charge.

### Statistical analysis

All collected data were statistically described and analysed. Absolute and percent frequencies were used for qualitative variables; mean, standard deviation (SD), range and median were used to summarize quantitative variables. Fisher’s exact test and chi-square test were used to compare the main variables distribution between treated and untreated children or subpopulations (Fisher’s exact test was used to compare sex, race, skin colour and income between treated and untreated children; chi-square was used to compare vitamin D status according to treatment or skin colour). t-test was used to explore the presence of differences in means of the main numerical variables between subpopulations (e.g. mean serum levels of 25-OH-D in age groups). Pearson’s correlation was used to verify the presence of linear correlations. The significance level of 0.05 (***P***) was used. SAS® software (SAS Institute Inc., Cary, North Carolina, USA) was used for statistical analysis.

25-OH-D levels of groups A and B were compared month to month and quarter to quarter to exclude seasonal effects [6, 9].

### Sample size calculation

The minimal sample size for each group was calculated 58. Considered parameters: a) an increase of at least 10 ng/ml in serum 25-OH-D levels; b) a population with a basal mean value 20.0 and SD 16.6 ng/ml [8]; c) a significance level ***P*** = 0.05 and a power of 90%.

## Results

### Demographic and physical characteristics

Between November 2010 and January 2015, serum 25-OH-D level was measured in 203 children. Active group A (***n*** = 82) were given 1500 IU vitamin D3/day from November to April; control group B (***n*** = 121) had not received any vitamin D supplementation over the last 12 months. About 25% of the children were first generation immigrants. See Table 1 for the full description of the demographic and physical characteristics and the ethnic make-up of groups A and B.

**Table 1:**
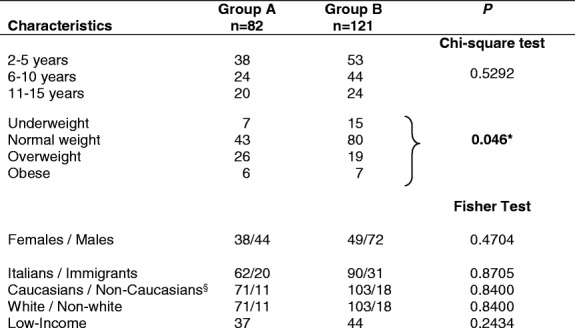
Demographic and physical characteristics of children (***n*** = 203)

^§^ Group A non-Caucasians 11: North Africans 6, Latin Americans 3, and sub-Saharan Africans 2. Group B non-Caucasians 18: North Africans 12, Asians 3, Latin Americans 2, and sub-Saharan African 1

**n** *= number of children in each group*

*below ***P*** *significance level*

### Prevalence of Hypovitaminosis D

25-OH-D varied from 8 to 71 ng/ml in group A and from 2 to 56 ng/ml in group B; the mean level in groups A and B was 31.9 ng/ml (SD = 13.0 ng/ml; interquartile range 23–38 ng/ml) and 21.8 ng/ml (SD = 10.1 ng/ml; interquartile range 16–27 ng/ml), respectively (***P*** <  0.001). Only 19.0% of group A had a normal level vs. 46.3% of group B. In group A 12.2% had vitamin D deficiency and 1.2% had severe vitamin D deficiency. In group B 46.3% had vitamin D deficiency and 9.1% had severe vitamin D deficiency, these differences being statistically significant (***P*** < 0.001): see Table 2. The datasets concerning children of group A and group B are presented in tabular form in Additional files 1 and 2 respectively.

**Table 2:**
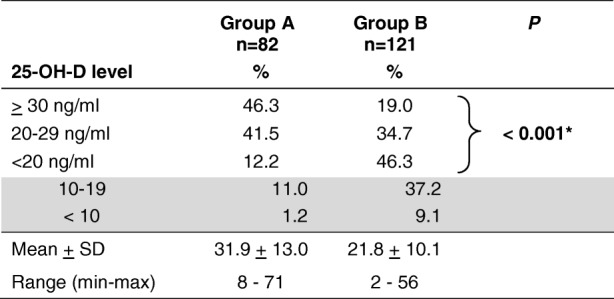
Distribution of children per 25-OH-D level

**n** = number of children in each group; * Chi-square test

### High 25-OH-D levels

With regard to the upper levels, in active group A 6% had 25-OH-D > 56 ng/ml (the maximum level reached by group B), 5% had a 25-OH-D level > 60 ng/ml and the maximum 25-OH-D level was 71 ng/ml. There were no side effects.

### Age effect of vitamin D3 supplementation

In control group B preschoolers, schoolers and adolescents had similar mean 25-OH-D levels around 21–22 ng/ml. In group A only preschoolers and schoolers had a statistically significant higher mean value in comparison to the same age of group B both of them above 30 ng/ml (Table 3). In adolescents 1500 IU vitamin D3/day caused only a slight increase of mean 25-OH-D level in the range of insufficiency, although the deficiency rate passed from 54 to 5%, with normal and insufficient children 25 and 70%, respectively (data not shown in the Table). For every supplementary 100 IU/day we found a rise of serum 25-OH-D of 0.93, 0.59 and 0.33 ng/ml in preschoolers, schoolers and adolescents, respectively (Table 3).

**Table 3:** Mean serum levels of 25-OH-D (ng/ml) per age group

		Total	Groups		Rise (ng/ml)	**T-test**
			A. Suppl.	B. No suppl.	every extra 100 IU/day	***P***
2–5 years	n	91	38	53		
	Mean ± SD		35.2 ± 14.1	21.3 ± 11.4	0.93	< 0.0001*
6–10 years	n	68	24	44		
	Mean ± SD		31.1 ± 12.9	22.3 ± 8.6	0.59	0.0013*
11–15 years	n	44	20	24		
	Mean ± SD		26.8 ± 8.8	21.8 ± 9.8	0.33	0.0850

n *= number of children in each group*

*below ***P*** *significance level*

### Vitamin D status according to skin colour

In control group B white children had a significantly higher mean 25-OH-D level in comparison to non-white children (23.1 + 9.8 vs. 14.2 + 7.9 ng/ml; ***P*** = 0.001). This difference was not present in group A (white 31.7 + 11.7 vs. non-white 33.7 + 19.8 ng/ml). However, non-white children had a higher vitamin D deficiency probability in comparison to white children (three times in group A: 27.3% vs 9.9%; double in group B: 77.8% vs. 40.8%): see Table 4.

**Table 4:** Vitamin D status according to skin colour

	Group A n = 82		Group B n = 121	
	Non-white n = 11	White n = 71	Non-white n = 18	White n = 103
25-OH-D level	**%**	**%**	**%**	**%**
> 30 ng/ml	45.5	46.5	–	22.3
20–29 ng/ml	27.3	43.7	22.2	36.9
< 20 ng/ml	27.3	9.9	77.8	40.8
All	100.0	100.0	100.0	100.0
**Chi-square test (** ***P*** **)**	0.225		0.009*	

n = number of children in each group; * below ***P*** *significance level*

None out of the 18 group A non-white children had 25-OH-D > 30 ng/ml. Among non-white children in group B, Africans had the lowest mean value (13.0 + 7.7 ng/ml).

### Period of blood withdrawal

The analysis of mean 25-OH-D levels per month is represented in Table 5.

**Table 5:** Mean serum levels of 25-OH-D (ng/ml) per month

		Total	Groups		**T-test** ***P***
			A. Suppl.	B. No suppl.	
01. January	n	29	14	15	0.009*
	**Mean**	21.6	**26.6**	**16.9**	
02. February	n	24	11	13	0.007*
	**Mean**	27.8	**36.0**	**20.8**	
03. March	n	16	8	8	0.014*
	**Mean**	25.5	**36.5**	**14.5**	
04. April	n	16	2	14	0.046*
	**Mean**	18.6	**30.5**	**16.9**	
05. May	n	23	8	15	0.861
	**Mean**	24.8	**25.4**	**24.5**	
06. June	n	21	10	11	0.096
	**Mean**	23.8	**28.6**	**19.4**	
07. July	n	16	9	7	0.627
	**Mean**	29.4	**30.7**	**27.9**	
08. August	n	12	7	5	0.574
	**Mean**	36.8	**38.4**	**34.6**	
09. September	n	10	3	7	0.295
	**Mean**	30.4	**36.0**	**28.0**	
10. October	n	10	4	6	0.049*
	**Mean**	29.2	**38.3**	**23.2**	
11. November	n	12	3	9	0.476
	**Mean**	26.0	**35.0**	**23.0**	
12. December	n	14	3	11	0.228
	**Mean**	26.3	**32.7**	**24.5**	

n *= number of children in each group*

*below ***P*** *significance level*

Group A had a higher mean 25-OH-D level than group B also outside the supplementation period. Children receiving supplementation showed normal to near-normal 25-OH-D levels in all months except in May. Group B demonstrated deficiency levels in 4 out of the first 6 months of the year and insufficiency in the other 2 months.

Children without supplementation showed a normal or near-normal mean level only in the third quarter of the year (mean level from July to September: 29.7 ng/ml).

**Fig. 1:**
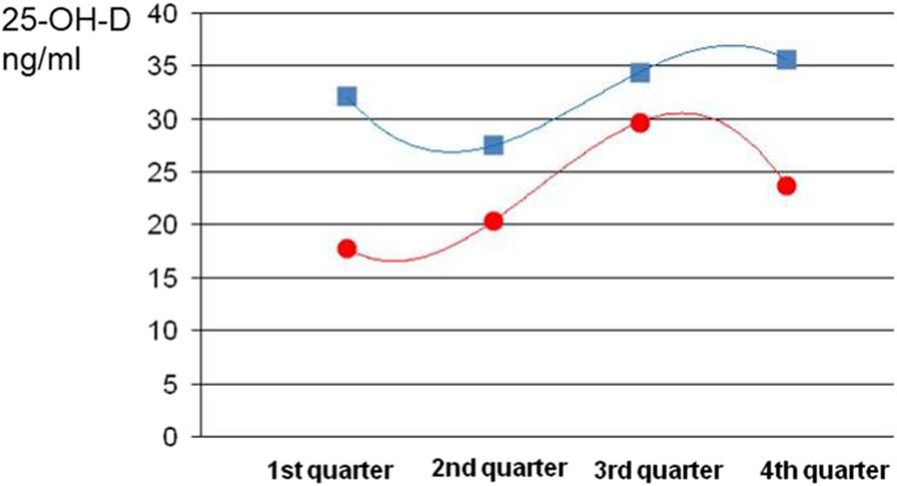
25-OH-D level according to period of blood withdrawal. Blue line: quarterly trend in supplemented children. Red line: quarterly trend in not supplemented children

The seasonal effect on 25-OH-D levels is represented in Fig. 1 and Table 6.

**Table 6:** Serum levels of 25-OH-D (ng/ml) per quarter

	1st Quarter		2nd Quarter		3rd Quarter		4th Quarter	
	January–March		April–June		July–September		October–December	
	Group A	Group B	Group A	Group B	Group A	Group B	Group A	Group B
n	33	36	20	40	19	19	10	26
Mean	**32.1**	**17.8**	**27.5**	**20.4**	**34.4**	**29.7**	**35.6**	**23.7**
SD	14.7	6.8	11.0	11.0	10.2	11.6	14.6	7.6
Median	28.0	19.0	27.0	18.0	35.0	29.0	29.5	24.5
Range	11–71	8–37	8–61	2–56	18–56	8–50	17–62	10–41
Interq. range	21–42	12.5–21	23–30.5	15–25.5	28–42	21–40	27–42	16–29
**T-test (** ***P*** **)**	< 0.001*		0.023*		0.195		0.033*	

**n** *= number of children in each group; SD = standard deviation*

*below ***P*** *significance level*

In sunny months mean solar radiation in the four consecutive years of observation was: 22.8 MJ/m2 in 2011, 21.7 in 2012, 21.3 in 2013 and 20.8 in 2014. Spring solar radiation has been relatively stable in these four years except for a slight increase in 2011: 24.2 MJ/m2 in 2011, 21.9 in 2012, 21.2 in 2013 and 22.6 in 2014; summer solar radiation has been relatively stable except for a slight decrease in 2014: 21.4 MJ/m2 in 2011, 21.5 in 2012, 21.4 in 2013 and 19.0 in 2014.

### Follow-up of group a

All children in group A were followed up: during the last 12 months prior blood withdrawal they had 1–21 visits (7,1 + 4.1 mean + SD; interquartile range 4–9) and continuing vitamin D3 supplementation was recommended. Nevertheless there was no linear correlation between number of visits and 25-OH-D levels (***P*** = 0.5928; *ρ* = − 0.05992).

Also children of group B had a similar follow-up.

## Discussion

DINOS was conducted in a paediatric primary care setting on 203 children living in a rural area of North Italy near Padova (45°N latitude). The 25% of immigrants in this study was representative of Veneto region (21.7% of births between 1999 and 2011) [47]. Group A children (***n*** = 82) received 1500 IU vitamin D3/day from November to April as seasonal supplementation; control group B (***n*** = 121) received no supplementation. Serum levels of 25-OH-D were measured in children of both groups; this metabolite is considered the best indicator of overall vitamin D status as it reflects total vitamin D from dietary intake and sunlight exposure, in addition to the conversion of vitamin D from adipose stores in the liver [34, 39, 48].

Our prevalence of 9.1% children with severe vitamin D deficiency is similar to those reported in other studies [8, 12, 15]. It could lead to rickets or, more often, to slight hypovitaminosis D with negative health consequences [4, 49–51]. Pettifor has highlighted that also values of 25-OH-D > 10–15 ng/ml can be associated with rickets when calcium intake is poor [52]. In DINOS it is remarkable that four out of five children in group B had hypovitaminosis D and over 46% had serum 25-OH-D < 20 ng/ml, which is the cut-off mostly associated with vitamin D deficiency [17, 20, 35, 37, 48, 53]. Similar prevalences of serum 25-OH-D < 20 ng/ml were reported in a sample of healthy US adolescents [14] and in two Italian studies [8, 12], where non-white children had a higher deficiency risk in comparison to whites, in agreement with our study results.

The major source of vitamin D for humans is the conversion of 7-dehydrocholesterol into previtamin D3 in the skin through sun exposure [43]; a further thermic conversion produces vitamin D3. In the autumn and winter months the skin generates little to no vitamin D at latitudes above 37° N and under 37°S *(vitamin D winter area*) [54]; just 2° under 45°N (the latitude of our area) no vitamin D is produced during late autumn, winter, and the beginning of spring [55]. Given these seasonal data, we could compare the two groups in different timespans because at 45°N sun exposition is in any case ineffective. With regard to sunny months we must consider that our area is near the sea with a moderate climate and only small fluctuations in seasonal weather, as shown by solar radiation data.

We found that children without vitamin D3 supplementation had a mean level of 25-OH-D < 20 ng/ml in 4 out of the first 6 months of the year and insufficient in the other two months; only in late summer they reached serum 25-OH-D maximum levels about 30 ng/ml. This value may be considered a “natural” level. Also a prolonged or excessive exposure to sunlight does not produce a toxic amount of vitamin D3 because pre-vitamin D3 is rapidly photodegraded to a variety of inactive products and 25-OH-D levels naturally self-regulate [56]. These data suggest that in the absence of valid sun exposure a supplementation is necessary.

In a previous report [9] we have demonstrated that factors consistently associated with a low vitamin D status include non-white skin colour. In our DINOS Study none out of 18 non-white children without vitamin D supplementation had 25-OH-D > 30 ng/ml, in agreement with the literature [8, 12].

Some studies have demonstrated that overweight and obese children are at risk of hypovitaminosis D [57, 58]. In our research children receiving vitamin D3 supplementation had higher levels of 25-OH-D in spite of a significant tendency for being overweight (the composition of groups A and B did not differ except for BMI distribution).

Other factors (e.g. diet and estimated sunlight exposure) were not analysed, However, none of the children were vegetarian and in Italy shelf food is generally not fortified with vitamin D. Furthermore, it is well known that vitamin D status depends minimally on food intake: only 10% of the daily vitamin D requirement is provided by food [59], in the form of vitamin D2 (ergocalciferol, from plants, especially sun-exposed mushrooms) and vitamin D3 (cholecalciferol, from dairy products and fatty fish). For sunlight exposure we considered that our population was rather homogeneous and that children of group A and group B have similar baseline characteristics (age, sex, ethnicity, skin colour and income). Moreover, an Italian study on a larger group of children has demonstrated that lifestyle has a low impact on 25-OH-D levels [11].

The supplementary vitamin D dose depends both on baseline and target serum level of 25-OH-D as well as BMI [11, 60, 61] and genetic factors [61].

There are a few studies on the dose impact of vitamin D supplementation in children. In most of them the rise of 25-OH-D serum levels for every extra 100 IU/day of vitamin D intake varies between 0.5 and 1.5 ng/ml in non-obese children (Table 7).

**Table 7:**
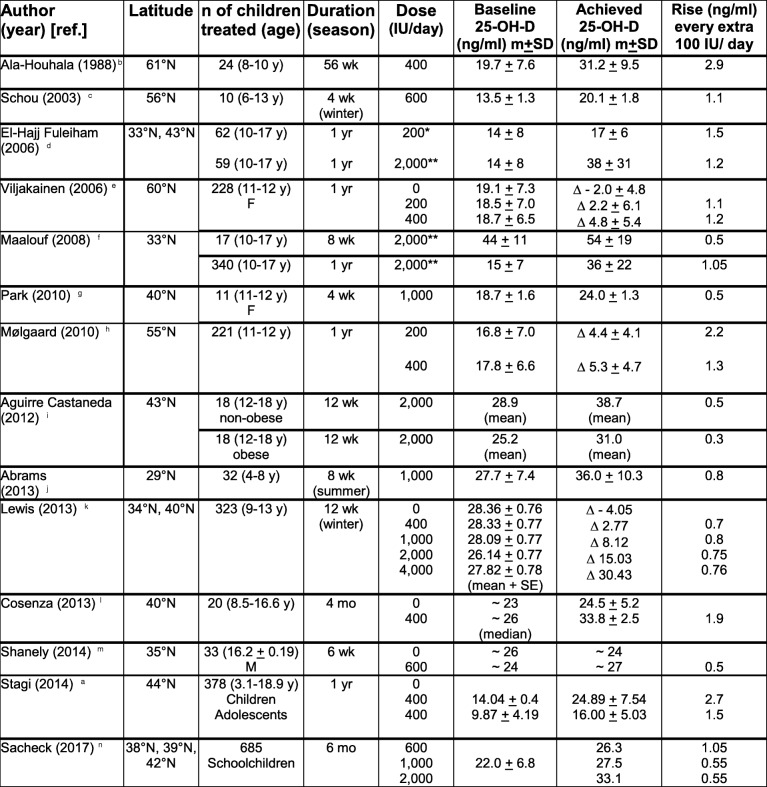
Vitamin D supplementation: randomised controlled trials with children

*Doses equivalent to 200 IU/day (1400 IU/wk)

**Doses equivalent to 2000 IU/day (14,000 IU/wk)

*F* females, *M* Males

^a^Stagi [11]

^b^Ala-Houhala [25]

^c^Schou [26]

^d^El-Hajj Fuleiham [27]

^e^Viljakainen [28]

^f^Maalouf [29]

^g^Park [30]

^h^Mølgaard [31]

^i^Aguirre Castaneda [32]

^j^Abrams [63]

^k^Lewis [67]

^l^Cosenza [68]

^m^Shanely [69]

^n^Sacheck [70]

Generally, lower baseline values of 25-OH-D correlate with a higher increase and vice versa [29, 57, 60]. Furthermore, for a given dose of vitamin D3 we found a higher increase of 25-OH-D in children than in adolescents, as reported by Stagi et al. [11].

NHANES 2001–2006 data have demonstrated that only 10% of children taking vitamin D supplements at doses 100–400 IU/day were deficient and over 50% were insufficient [2]. However, it must be taken into account that in North America food is often fortified with vitamin D. This practice is common also in North European countries but not in Italy.

At the request of the US and Canadian governments the IOM calculated that a Recommended Dietary Allowance (RDA) of 600 IU/day ensures a 25-OH-D serum level of 20 ng/ml to 97.5% of the population aged 1–18 years [20]. Recently, Veugelers and Ekwaru [62] noted that the IOM estimate of 600 IU vitamin D/day as RDA is incorrect because of a statistical error; these authors concluded that with the current recommendation of 600 IU/day, bone health objectives and disease and injury prevention targets will not be met. The same dose of 600 IU/day was recommended by the AAP [21] and Italian LARN [22].

There is accumulating evidence to suggest that vitamin D intake should be in the range of at least 800–1000 IU/day [59, 61, 63]. The evidence-based recommendations by the Endocrine Society’s Clinical Practice Guidelines to prevent vitamin D deficiency states 400–1000 IU/day supplementation for children [64] as does the Italian Health Minister [23]. The Italian 2015 Consensus recommends 600–1000 IU/day in children aged 1–18 years at risk of hypovitaminosis D and 2000 IU/day for 6–8 weeks in children and adolescents with 25-OH-D < 20 ng/ml [24]. This strategy presupposes that we know the level of 25-OH-D in each patient but measurement of the 25-OH-D concentration is not routinely recommended [24]. Also Holick said it could be more cost-effective to implement a vitamin D supplementation program for all children and adults rather than measuring 25-OH-D levels. The cost of a 25-OH-D test corresponds to that of a ten month prophylaxis with 1000 IU vitamin D3/day. In our opinion a possible solution could be to prescribe a high enough dose for all children to correct a potential vitamin D deficiency within the hypervitaminosis safety range.

In our experience a vitamin D3 supplementation with 1500 IU/day for six months ensured a 25-OH-D > 30 ng/ml in about 50% and > 20 ng/ml in 88% of children. Only 6% of children in group A had 25-OH-D > 56 ng/ml, which is the maximum level reached by the control group. The maximum 25-OH-D level in group A was 71 ng/ml, well away from the hypervitaminosis cut-off.

A vitamin D3 dose of 1500 IU/day, as in DINOS, is higher than the indication for Italian children [23], although below the upper safety level, but still 12% of active group A had vitamin D deficiency. Higher doses could be more appropriate, especially in adolescents. In fact for the same dose of vitamin D3 we found a higher increase of serum 25-OH-D in children (preschoolers and schoolers 0.93 and 0.59 ng/ml, respectively, every supplementary 100 IU/day of vitamin D3) than in adolescents (0.33 ng/ml). For adolescents in North Italy 2000 IU vitamin D3/day might be more appropriate, according to Holick’s recommendation of 1500–2000 IU/day from 11 to 19 years of age and 1000–1500 IU/day from 1 to 10 years [33]. Given the 0.33 ng/ml rise of 25-OH-D every extra 100 IU/day found in our adolescents aged 11–15 years (see Table 3), a dose of 2500 IU vitamin D3/day might be prescribed in this age group to reach the minimum desirable level of 25-OH-D. Adolescence, indeed, is a crucial phase for bone development when the most rapid bone accrual occurs [65].

The concept of *vitamin D winter area* is well known [54], but scientific societies guidelines seldom go into detail on this argument. The Canadian Pediatric Society identifies the period from October to March at the latitude of Edmonton (52° N) [37]. Central Europe guidelines recommend prophylaxis between September and April or throughout the whole year if sufficient skin synthesis of vitamin D is not ensured in the summer [41]. The Italian 2015 Consensus suggests prophylaxis between November and April in children with risk factors of hypovitaminosis D [24]. However, a research on hospitalized children in North Italy found a higher rate of hypovitaminosis D between November and May [6]. In DINOS children receiving supplementation showed normal to near-normal 25-OH-D levels in all months except in May. On this basis we suggest a supplement prolongation until May could be useful. More prolonged periods could be appropriate especially in non-white children. The French Society of Pediatrics recommends vitamin D prohylaxis in non-Caucasian children all through the year [66].

## Conclusions

This DINOS Study conducted on 203 children living in North Italy indicates that a daily supplementation with at least 1500 IU cholecalciferol from November to April is appropriate for most children in our region. A prolongation until May could be useful, as do higher doses and/or prolonged periods, especially in adolescents and in non-white children. The 25-OH-D level of 30 ng/ml reached in late summer by children not receiving vitamin D3 supplementation may be considered a “natural” level. Even if the results of our study are concerning North Italy, we think that the prevalence of hypovitaminosis and the effect of vitamin D supplementation are meaningful to others parts of the *vitamin D winter area* at the same latitude (45°N), from Ottawa (Canada) to Harbin (North China).

We invite for more research in this area. Prospective studies with larger numbers of subjects, possibly multicenter RCTs, are needed to further define vitamin D needs of all children living in North Italy and at the same latitude (45°N) in other countries and elsewhere in the world. It is of interest also to study the clinical advantage of chronic vitamin supplementation versus the burden of a continuous drug administration.

## Additional files

Additional file 1: Dataset of active group A (n = 82). (XLS 43 kb)

Additional file 2: Dataset of control group B (n = 121). (XLS 95 kb)

## Abbreviations

25-OH-D: 25-hydroxyvitamin D
AAP: American Academy of Pediatrics
BMI: Body mass index
EFSA: European food safety authority
IOM: Institute of medicine
LARN: Livelli di Assunzione di Riferimento di Nutrienti
NHANES: National Health and Nutrition Examination Survey
NHS: National health service
PTH: Parathyroid hormone
RCT: Randomized controlled trial
SD: Standard deviation
SIOMMMS: Società Italiana dell’Osteoporosi, del Metabolismo Minerale e delle Malattie dello Scheletro
WHO: World Health Organization
